# Accessory genes in tropical race 4 contributed to the recent resurgence of the devastating disease of Fusarium wilt of banana

**DOI:** 10.21203/rs.3.rs-3197485/v1

**Published:** 2023-08-08

**Authors:** Li-Jun Ma, Yong Zhang, Chunyu Li, Siwen Liu, Cunwu Liu, Diane Mostert, Houlin Yu, Sajeet Haridas, Katie Webster, Minhui Li, Igor Grigoriev, Altus Viljoen, Ganjun Yi

**Affiliations:** University of Massachusetts Amherst; University of Massachusetts Amherst; Institution of Fruit Tree Research; Institution of Fruit Tree Research; Institution of Fruit Tree Research; Department of Plant Pathology, University of Stellenbosch, Private Bag X1, Matieland 7602, South Africa; University of Massachusetts Amherst; Lawrence Berkeley National Lab; University of Massachusetts Amherst; South China Agricultural University; US DOE Joint Genome Institute/ Lawrence Berkeley National Lab/ University of California Berkeley; Department of Plant Pathology, University of Stellenbosch, Private Bag X1, Matieland 7602, South Africa; Institution of Fruit Tree Research

## Abstract

Fusarium wilt of banana, caused by *Fusarium oxysporum* f. sp. *cubense* (*Foc*), is one of the most damaging plant diseases recorded. *Foc* race 1 (R1) decimated the Gros Michel–based banana trade. Currently, tropical race 4 (TR4) is threatening the global production of its replacement cultivar, Cavendish banana. Population genomics and phylogenetics revealed that all Cavendish banana–infecting race 4 strains shared an evolutionary origin that is distinct from R1 strains. The TR4 genome lacks accessory or pathogenicity chromosomes, reported in other *F. oxysporum* genomes. Accessory genes—enriched for virulence and mitochondrial-related functions—are attached to ends of some core chromosomes. Meta-transcriptomics revealed the unique induction of the entire mitochondria-localized nitric oxide (NO) biosynthesis pathway upon TR4 infection. Empirically, we confirmed the unique induction of NO burst in TR4,suggesting the involvement of nitrosative pressure in its virulence. Targeted mutagenesis demonstrated the functional importance of accessory genes *SIX1* and *SIX4* as virulent factors.

The banana shortage referred to in the 1920s musical hit “Yes! We Have No Bananas” by songwriters Frank Silver and Irving Cohn was caused by Fusarium wilt of banana (FWB), one of the most severe epidemics in agricultural history^1^. The pathogen responsible for FWB is a fungus named *Fusarium oxysporum* f. sp. *cubense* (*Foc*), a member of the *F. oxysporum* species complex (FOSC). During the first half of the 20^th^ century, *Foc* race 1 (R1) destroyed millions of Gros Michel banana plants in Central America^2^. To ensure continued banana production, Gros Michel bananas were replaced with R1-resistant Cavendish bananas as the dominant cultivated variety. Yet, at the turn of 21^st^ century, a resurgence of FWB caused by a different strain, tropical race 4 (TR4), again decimated banana plants, particularly Cavendish bananas. Since its initial report in Taiwan in 1989^3^, TR4 has spread from its suspected origin of Indonesia and Malaysia to the Northern Territory of Australia, the Philippines and mainland China^4^. Recently, TR4 was detected in the Middle East (Oman, Jordan and Pakistan), Mozambique (2013), India (2015), Vietnam (2016), Laos (2016), Myanmar (2018) and Israel (2018)^5^. Its recent appearance in Columbia (2019)^6^ and Peru (2021)^7^, which together constitute the largest Cavendish banana–exporting region of the world, poses an imminent risk to the global banana supply. Apart from threatening the production of export bananas, TR4 infects locally consumed bananas, including other banana varieties. As bananas are a staple food and one of the main dietary sources of carbohydrates in Africa, Southeast Asia and tropical America, TR4 poses a major threat to both the global banana trade and to the food security of people living in these areas, having the potential to exacerbate poverty in developing nations and create food shortages that intensify world hunger^4^.

Members of the FOSC are responsible for wilt diseases in many economically important crops^8^. Thus, *F. oxysporum* is listed among the top five most important plant pathogens^9^. Horizontally inherited accessory chromosomes (ACs)—also referred to as pathogenicity, dispensable, or lineage-specific chromosomes—that are deficient in genes associated with housekeeping functions, but enriched in genes related to fungal virulence, determine the host-specific pathogenicity among FOSC members^10–14^.

Three *Foc* races have been distinguished based on their host-specific pathogenicity on certain banana cultivars. *Foc* strains are further divided into 24 vegetative compatibility groups (VCGs)^15–17^, reflecting the ability of strains within the same VCG group to fuse and form heterokaryons. Closely related *Foc* VCGs are grouped in phylogenetic lineages^15,18^. The pathogen that destroyed the Gros Michel banana industry belongs to *Foc* R1^19^. *Foc* race 2 (R2) affects ‘Bluggoe’ (ABB) bananas and other cooking bananas^20^. *Foc* TR4 is a member of *Foc* race 4 (R4), which also includes *Foc* subtropical race 4 (STR4) that infects Cavendish bananas grown in subtropical regions under sub-optimal conditions (cool temperatures, water stress or poor soil health). TR4 causes disease in banana plants grown in both the tropics and subtropics even in the absence of predisposition to stress factors^20^ (Fig. 1a). Most reported TR4 strains belong to the VCG 01213/16 complex. Strains belonging to the distinct VCG 0121 complex also cause disease in Cavendish bananas similar too TR4^4^.

The international research community has generated genomic resources that include the assemblies of UK0001^21^, TR4 58^22^ and PerS4 (Peru strain)^23^ and has re-sequenced *Foc* TR4 strains from Israel/Middle East^24^, Colombia^6^, Laos, Vietnam and Myanmar^25^. However, our understanding of the *Foc* evolutionary history and of the pathogenesis of TR4 remains limited. Here, we explored the genetic diversity among *Foc* strains with a focus on understanding the genetic basis of TR4 virulence. By comparing 35 *Foc* isolates covering 23 VCG groups, we confirmed thepolyphyletic nature of *Foc* and discovered that all R4 strains—TR4, STR4 and VCG 0121—share a single origin and that VCG 0121 and TR4 01213/16 VCG complexes may represent progenies of a mating population. High-quality TR4 genome assembly lacks independent accessory chromosomes but possesses accessory genes that are attached to the ends of several core chromosomes. The TR4 accessory genes are enriched for virulence and mitochondria-related functions. Based on our genomics, transcriptomics and pathogenicity studies, we established that the mitochondria-localized fungal nitric oxide (NO) biosynthesis pathway is involved in the TR4–banana interaction. Biochemical and molecular characterization as well as reverse genetics confirmed that the acquisition of accessory genes contributes directly to TR4 pathogenesis.

## Results

### 1. Foc strains are diverse and R4 strains form a monophyletic clade.

**Figure 1:**
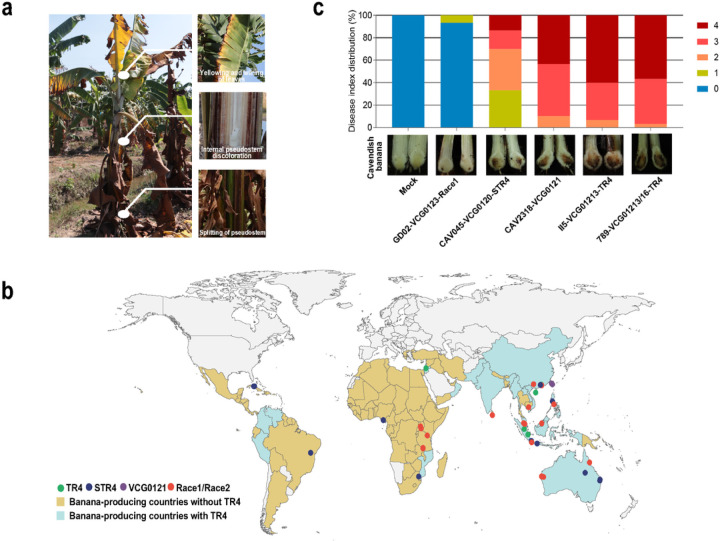
The most virulent *Foc* variant (TR4) has spread from Southeast Asia to Africa and Latin America. **a)** Disease symptoms of Fusarium wilt of banana caused by TR4. Affected plants wilt rapidly. Yellowing and wilting of the leaves progress from the older to the younger leaves, and plants eventually die with pseudostem splits. Internally, vascular discoloration occurs in the roots and rhizomes with a yellowing that proceeds to a reddish-brown color in the pseudostem. **b)** Global distribution of TR4 and geographical origins of all 35 strains included in this study. Different races are represented by different colored dots: green dots, *Foc* TR4 strains; blue dots, *Foc* STR4 strains; purple dots, *Foc* VCG0121 strains; and red dots, *Foc* R1 and R2 strains. **c)** Disease severity of Cavendish banana with four or five leaves inoculated with mock (H_2_O) and conidia (5000 conidia/g soil) of R1 strain GD02 (VCG0123), STR4 strain CAV045 (VCG0120), VCG0121 strain CAV2318, and two TR4 strains, II5 and 789 (VCG01213/16). The disease index (DI) distribution (top panel, details see methods section) was calculated among 20 inoculated plants based on the disease index from 0–4: 0, no symptoms; 1, some brown spots in the inner rhizome; 2, <25% of the inner rhizome showing browning; 3, up to 75% of the inner rhizome showing browning; and 4, 100% inner rhizome and pseudostem appearing dark brown (considered dead). The cross-sections of inoculated plantlets (bottom panel) were sampled at 30 days post inoculation. The extend of discoloration is correlated with the DI distribution shown in the top panel.

To trace the evolutionary history of *Foc* strains, we conducted a comprehensive population genomics study using *Foc* strains that were collected from banana production locations around the world and represent all three races in the 23 *Foc* VCG groups (Fig. 1b). The 35 selected *Foc* genomes comprise 5 TR4, 12 STR4, 2 VCG 0121 and 16 R1 and R2 strains. With the genetic hallmarks of a heterothallic sexual life cycle, a *Foc* genome has either a *MAT1–1* or *MAT1–2* gene at the mating locus. Strains within the same VCG shared the same mating-type idiomorph, and *MAT1–1* and *MAT1–2* were present in all three *Foc* races (Supplementary Table 1). Pathogenicity tests confirmed that R1 showed limited symptoms in Cavendish bananas, STR4 strains caused mild disease symptoms, and TR4 and VCG 0121 strains induced more severe disease symptoms than the other strains (disease index [DI]: R1 = 0.15; STR4 = 0.2; VCG 0121 = 3.15; TR4 = 3.2) (Fig. 1c and Supplementary Table 2).

**Figure 2:**
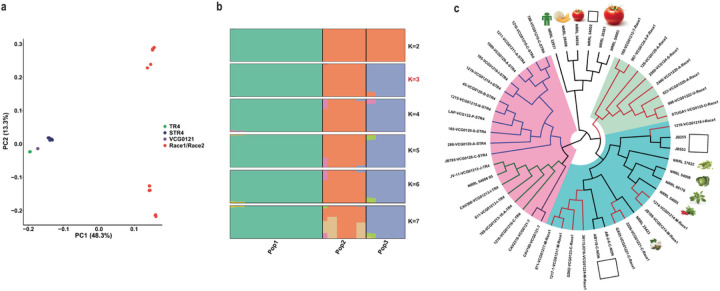
*Foc* phylogenomics and population structure. **a)** Separation of individual strains by PCA using genetic variants from SNP calling. Three genetic clusters were projected by principal components PC1 and PC2. Numbers in parentheses represent the percentage of total variance explained by the first and second PCs. Different races are presented by different colored dots: green, *Foc* TR4; blue, *Foc* STR4; purple, *Foc*VCG0121; and red, *Foc* R1 and R2. **b)** The population structure of all *Foc* isolates was analyzed using the admixture model (10,000 iteration burn-in and 10,000 iteration) in software STRUCTURE based on whole-genome SNPs. Structure was run for K values between 1 and 7 without prior population information. The best K value, 3, was selected by the Evanno method in STRUCTURE HARVESTER. Each isolate is represented by a bar, and the length of each colored segment in each bar represents the proportion contributed by ancestral populations. Distinct from *Foc* R1 strains, all Cavendish banana–infecting *Foc* R4 strains forming a unique population, pop1. **c)** Phylogenetic status of *Foc*and selected *F. oxysporum* strains. The phylogeny was inferred from the maximum likelihood analysis of 10 conserved single-copy orthologous genes within the *Fusarium* genus. The color scheme of branches is as in panel **a.** The pink shaded region highlights pop1, including all Cavendish banana–infecting *Foc* R4 strains. The blue shaded region highlights pop2 and several other *F. oxysporum formae speciales*. The green shaded region highlights pop3.

We identified 2,021,590 SNPs, accounting for ~4% of the genome, across the 35 *Foc* isolates when compared to the high-quality reference genome *Foc* TR4 II5 (NRRL 54006) (https://mycocosm.jgi.doe.gov/FoxII5/FoxII5.home.html). Principal component analysis (PCA) revealed a well-defined structure containing three distinct populations, with all R4 strains—TR4, STR4 and VCG 0121 —grouping into one population and strains from R1 and R2 grouping into two populations (Fig. 2a). This structure was independently inferred using *STRUCTURE* software (Fig. 2b), with Pop1 containing only R4 strains, Pop2 containing six R1 VCGs (VCGs 0123, 01214, 01217, 01218, 01221 and 01224) and Pop3 containing six VCGs from both R1 and R2 (VCGs 0124, 0125, 0128, 01220, 01212 and 01222). *Foc* diversity was further assessed within the FOSC using 10 conserved single-copy orthologs^10^. In agreement with the population genomics results, all *Foc* strains formed three major clades (Fig. 2c). Pop2 and Pop3 were closely related to strains from other hosts, while all R4 strains of Pop1 formed a monophyletic clade, suggesting that *Foc* R4 has an independent origin compared to other strains in the FOSC (Fig. 2c).

### 2. The TR4 reference genome lacks accessory chromosomes.

**Figure 3:**
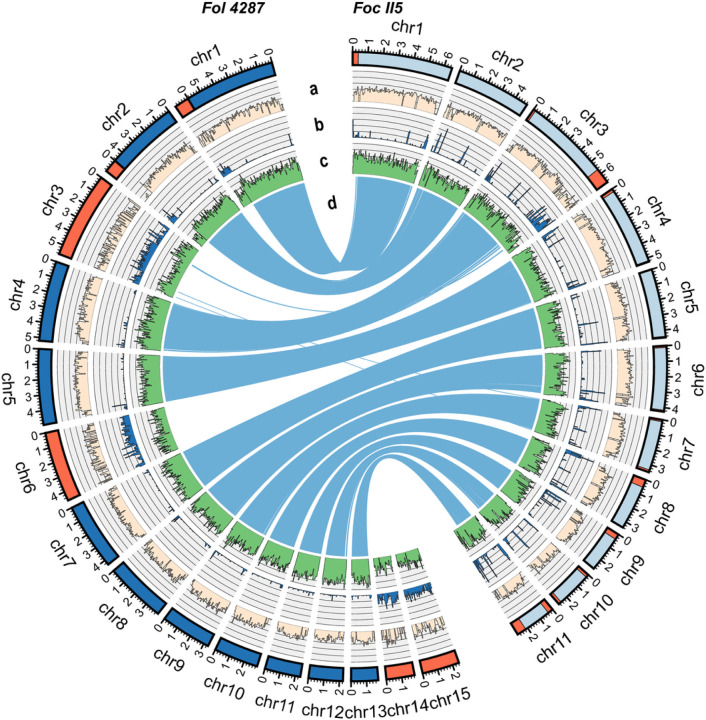
Whole-genome comparison between *Foc* II5 and *Fol* 4287 reveals the existence of accessory sequences in core chromosomes. The *Foc* II5 genome has 11 conserved core chromosomes (light blue) and accessory sequences (orange) but lacks conventional accessory chromosomes. The accessory sequences typically have a low GC content (a), high repetitive sequence composition (b), and low gene density (c). The syntenic alignment between *Foc* II5 and *Fol* 4287 using Nucmer (d) indicates the core chromosomal extended regions lacking synteny between *Foc* II5 and *Fol* 4287. The remaining alignments are shared mostly between core regions in both genomes.

TheTR4 II5 (NRRL 54006) genome was assembled into 11 core chromosomes. Distinct from other reported plant pathogenic *F. oxysporum* genomes, which all contain independent ACs^11,26^, the II5 genome lacked ACs, but carried a total of 4.84-Mb accessory sequences, primarily located at the ends of chromosome 3 (1.19 Mb) and chromosome 11 (0.97 Mb) (Supplementary Table 3, Fig. 3). This chromosome 3 architecture was preserved among five sequenced *Foc* TR4 genomes with the exception of the genome of strain UK0001 (Extended Data Fig. 1a)^21^, where we detected a recent chromosomal translocation between chromosomes 3 and 11 facilitated by an active transposable element, Helitron^27^ (Extended Data Fig. 1a) (Supplementary Table 4).

To trace the evolutionary footprints among *Foc* strains, we generated high-quality genome assemblies for STR4 (CAV 045), R1 (GD02) and VCG 0121 (CAV 2318) (Table 1), all of which have 11 core chromosomes (Extended Data Fig. 1). Using Benchmarking Universal Single-Copy Orthologs (BUSCO v3.1), we detected 99.3–99.6% of conserved fungal genes in these assemblies and confirmed their completeness (Table 1). Consistent with their phylogenetic relatedness (Fig. 2) and similarity in disease severity (Fig. 1c), the VCG 0121 genome had more II5 accessory sequences (3.36 Mb, 69.4%) than the STR4 (2.16 Mb, 44.6%) and R1 (1.58 Mb, 32.6%) genomes (Supplementary Table 5). The genomes of VCG 0121 and II5 also shared the chromosome 3 architecture, including the almost identical extended accessory sequences with 99.8% sequence identity covering 67% of the 1.1-Mb region (Extended Data Fig. 1c). However, VCG 0121 strains carried the *MAT* 1–2 idiomorph, whereas the TR4 strains all carried the mating-type locus *MAT* 1–1 (Supplementary Table 1). These observations suggest that VCG 0121 and the TR4 01213/16 VCG complex may represent progenies of a mating population in which one of the parental strains already carried the chromosome 3 architecture. The distribution of SNPs in VCG 0121 also suggests that a potential mitotic recombination event occurred before the split of the VCGs 01213/16 and 0121 (Extended Data Fig. 2). Collectively, our data indicate that sexual reproduction may have occurred right before the recent TR4 clonal expansion.

### 3. Nitric oxide (NO) is produced in Foc TR4 mitochondria soon after infection.

To better understand *Foc*–banana interactions, we compared meta-transcriptomics of Cavendish bananas infected with R1 (GD02) and TR4 (II5). Three infection time points were chosen, i.e., 18, 32, and 56 hours post-inoculation (HPI), representing the three critical biological states of the pathogen penetrating through, spreading within and becoming dominant in the host tissues (Extended Data Fig. 3a) (Supplementary Table 6). The read mapping matrix revealed a steady increase of fungal biomass over time: from 2.04 to 5.84 to 31.10% for R1-infected banana and from 11.82 to 34.68 to 56.85% in TR4-infected banana (Extended Data Fig. 3b), reflecting the increased aggressiveness of the TR4 pathogen (Extended Data Fig. 3b and Supplementary Table 6). The data also suggested that Cavendish bananas are not completely immune to R1.

Using a global hierarchical clustering algorithm, we identified 18 *Foc* co-expressed gene clusters among 12,235 genes that were expressed in both R1 and TR4 strains (Extended Data Fig. 4). The Pearson’s correlation coefficients (PCCs) comparing banana infected with either R1 or TR4 increased over time, with values of 0.74, 0.83 and 0.9 at 18, 32 and 56 HPI, respectively (Extended Data Fig. 3c), suggesting that the most distinct transcriptional reprogramming occurred at 18 HPI. Focusing on genes that were activated at this time point, we identified three fungal gene clusters, *Foc*-C5, *Foc*-C7 and *Foc*-C14, comprising 2,050 genes, that were significantly induced at 18 HPI in TR4 compared to R1 (Extended Data Fig. 5a). Among these genes, those encoding mitochondrial envelope–localized proteins were significantly enriched (corrected *p*-value = 0.0005). Specifically, *Foc*-C5 was enriched for electron transfer activity (corrected *p*-value = 0.01), *Foc*-C7 for heme-copper terminal oxidase activity (corrected *p*-value = 0.03) and *Foc*-C14 for NADPH quinone reductase activity and regulation of the nitrogen compound metabolic process (corrected *p*-value = 0.05) (Extended Data Fig. 5b).

**Figure 4:**
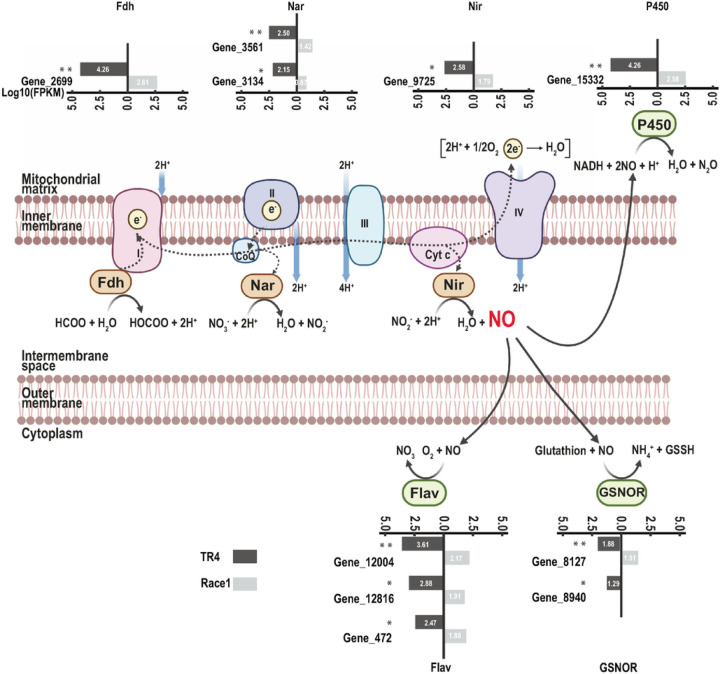
Mitochondria-localized nitrate/nitrite-dependent nitric oxide (NO) biosynthesis pathway and NO detoxification–related genes are significantly induced in TR4 at 18 HPI. Nitrate/nitrite-dependent NO biosynthesis pathway and NO detoxification–related genes are highlighted in light brown and green, respectively. Electron flow is represented by a dashed arrow. Expression patterns of orthologous genes at 18 HPI are presented side-by-side, *Foc* TR4 (dark gray) and *Foc* R1 (light gray), as values of log_10_(FPKM). All genes are significantly induced in TR4 at 18 HPI (** = *p-value* < 0.001, * = *p-value* < 0.01) according to the Student’s *t* test. The same patterns of expression were also observed for genes constituting the electron transport chain complexes, labelled as I–IV (See supplementary table S7). Fdh, NAD(+)- dependent formate dehydrogenase; Nar, nitrate reductase; Nir, nitrite reductase; P450, cytochrome P450; Flav, flavohaemoglobin; GSNOR, S-nitrosoglutathione reductase; CoQ, quinone; Cyt c, cytochrome c.

All genes functioning in the mitochondria-localized nitrate/nitrite-dependent pathway for fungal NO biosynthesis^28^ were expressed at significantly higher levels in TR4 strains than in R1 strains (Fig. 4). For example, Gene_2699 (NAD(+)-dependent formate dehydrogenase) and Gene_9725 (nitrite reductase) showed 40-fold and 6-fold higher expression in TR4 compared to R1 at 18 HPI, respectively (Supplementary Table 7). Similarly, the NO detoxification–related genes, which encode proteins such as flavohaemoglobin, cytochrome P450 and GSNO reductases that help pathogens protect themselves against nitrosative stress, were also uniquely induced in TR4-infected banana plants (Fig. 4, Supplementary Table 7).

The involvement of a potential NO burst was also supported by the significantly up-regulated banana phytoglobin genes, which are regulated by NO level and serve as active scavengers of NO. One of them, Ma02_g10610, was detected among top 20 most significantly induced genes upon TR4 inoculation (Supplementary Table 8). qRT-PCR analysis confirmed that all three banana phytoglobin homologs were highly induced at 18 HPI upon TR4 inoculation (Supplementary Table 8, Extended Data Fig. 6). These up-regulated phytoglobin genes belong to banana transcriptional expression cluster plant-C22, which contains 1,022 genes with similar expression patterns (Supplementary Table 9, Extended Data Fig. 7). Interestingly, plant-C22 also included two jasmonic acid (JA) biosynthesis-related genes, *OPR2* (Ma03_g02640) and *OPR3* (Ma07_g02270) (Supplemental Table 10), encoding the 12-oxo-phytodienoic acid reductases (OPRs).

**Figure 5:**
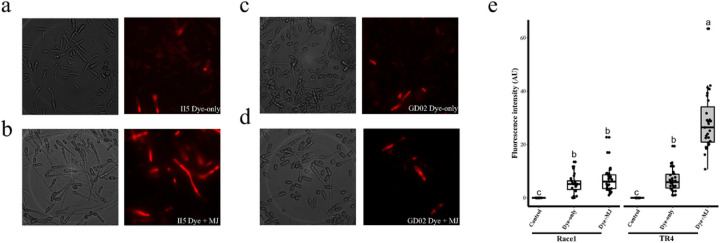
Plant hormone induced fungal NO burst in TR4. The comparison of fungal NO production in TR4 strain II5 and R1 strain GD02 was performed using the NO-sensitive fluorescent dye DAR-4M-AM as a probe. Methyl jasmonate (MJ), one of the jasmonic acid forms that functions as the phytohormone dispersed in plants, was used as the treatment. The fluorescence signal corresponding to the NO production was detected by confocal microscopy. The mock solution contained the fluorescent dye DAR-4M-AM only and the treatment solution contained both the fluorescent dye and 5 µM MJ. Fungal conidia were cultured in a solution for 12 hours before measure the fluorescence intensity. **a).** Mock-treated TR4 strain II5; **b)**MJ-treated TR4 strain II5. **c)**. Mock-treated R1 strain GD02; **d)** MJ-treated R1 strain GD02. **e)** Fluorescence intensity measuring NO production by fungal conidia. In the absence of the NO-sensitive fluorescent dye, no fluorescence signal was detected. In mock-treated samples, we detected comparable levels of NO signals for TR4 strain II5 and R1 strain GD02. The level of fluorescence showed significant increase in TR4 strain II5 in responding to MJ treatment. Data are shown as box plots with the interquartile range as the upper and lower confines of the box, and the median as a solid line within the box. Different letters indicate statistically significant differences according to the one-way ANOVA test (*p* < 0.05).

JA is a primary defense hormone and is involved in *F. oxysporum*–tomato^29^ and *F. oxysporum*–Arabidopsis^30,31^ interactions. Based on our transcriptomic analysis, we hypothesized that the NO burst in fungi during the TR4–banana interaction, which disarms plant immunity, was induced by the initial plant defense response involving plant JA biosynthesis. To test this hypothesis, we compared *in vivo* NO production betweenTR4 and R1 using the NO-sensitive fluorescent probe DAR-4M-AM^32^ upon JA stimulation (Fig. 5). With a sensitivity level of 0.1 mM nitroprusside^33^, we detected comparable levels of NO signal in both strains. However, we observed a significant fluorescent signal burst only in TR4 in response to JA signaling, as the average cytoplasmic fluorescence intensity in MJ-treated TR4 was about 4.5 times higher than that of non-treated cells and 4.2 times higher than that of R1 cells treated with MJ. All evidence points to the direct involvement of fungal NO in TR4 pathogenesis.

### 4. TR4 accessory genes contribute to mitochondrial activities and host pathogenicity.

To examine genetic components that contribute to TR4 virulence against Cavendish banana plants, we identified 1,587 TR4 encoding accessory genes. Interestingly, these genes are significantly enriched for mitochondrial functions (*p <* 0.05), including NADH dehydrogenase (ubiquinone) (*p*-value = 0.00003), electron transfer (*p*-value = 0.019) and biological processes involved in ATP synthesis (*p*-value = 0.007) (Supplementary Table 11). More than half of the TR4 accessory genes (856), also enriched for mitochondrial functions, were expressed during fungal infection (Supplementary Table 12). For instance, 38 expressed II5 accessory genes were significantly enriched for electron transport (*p*-value = 0.05) and 6 were targeted to mitochondria (Supplementary Table 13), including two as parts of the fungal NO biosynthesis pathway. The overlap between accessory genes and induced gene functions supports the notion that TR4 accessory genes contribute directly to the pathogen’s ability to impose nitrosative stress to disarm host defense.

Transcriptional regulation was another significantly enriched functionality among expressed accessory genes (*p*-value = 0.0098), including transcription factor (TF) genes encoding the GAL4-like Zn(II)2Cys6 binuclear cluster DNA-binding domain (Supplementary Table 12). In the II5 genome, this TF family was expanded to 630 members, which is substantially more than those identified in *Saccharomyces cerevisiae* (37), *Neurospora crassa* (114), *Magnaporthe oryzae* (143), *Aspergillus nidulans* (257) and *Fusarium graminearum* (263). Among the expanded TF gene family, 26 accessory genes, along with the core Gene_7374, were grouped with *FgZC1* (FGSG_05068) (Extended Data Fig. 8), a transcription factor encoding gene required for host-mediated fungal NO production and virulence in *F. graminearum*^34^. At 18 HPI, the expression of Gene_7374 was 3-fold higher in TR4 compared to its orthologous gene in R1. Three expression patterns were identified among these 26 accessory genes: 7, 11 and 8 genes were induced at 18, 32 and 56 HPI, respectively (Supplementary Table 14). In addition, this TF gene family included many genes encoding transcription regulators, such as *FoFow2*, which controls the plant infection capacity of *F. oxysporum*^35^; *FoEbr1*, which regulates genes involved in general metabolism and virulence^36^; and *FoFtf 1*, which regulates virulence and the expression of *SIX* effectors^37^.

In addition to confirming that the acquisition of accessory genes may have enabled TR4 to produce a NO burst upon encountering the host, we identified 61 small secreted fungal effectors among the proteins encoded by the expressed TR4 accessory genes (Supplementary Table 15). These effectors included seven that were secreted in xylem and encoded by *SIX* genes^38^, including three *SIX1* genes, one *SIX4* gene, one *SIX8* gene and two *SIX9* genes. Six out of seven *SIX* genes were in the accessory regions of chromosome 3 (Supplementary Table 15).

We conducted functional characterization of *SIX1* and *SIX4* due to the unique expansion of *SIX1* in TR4 and the unique expression pattern of *SIX4* upon infecting the host banana. Even though *SIX4*was present in most *Foc* genomes, its expression was only detected in TR4 (Supplementary Table 15). In contrast to the other *SIX* genes that were highly expressed at 56 HPI, *SIX4* was the only one that was highly expressed at 18 HPI (Supplementary Table 15). *SIX1a* and *SIX4* gene knockout mutants (*Dsix1a* and *Dsix4*) exhibited significantly reduced virulence in banana (*p* < 0.01) (Extended Data Fig. 9), further demonstrating that the accessory sequence regions of *Foc* TR4 are involved in fungal virulence in Cavendish banana.

## Discussion

Combining population genomics and phylogenetics techniques, this study inspected 35 *Foc* genomes representing 23 *Foc* VCG groups and all three *Foc* races within the FOSCframework and revealed a defined population structure. Whereas historic R1 and R2 strains are phylogenetically related to other *F. oxysporum formae speciales*, all *Foc* R4 strains—TR4, STR4 and VCG 0121—form a distinct population or phylogenetic clade, supporting the idea that R4 strains share an evolutionary origin^17^.

We carefully inspected the genome assemblies of severalR4 strains and observed that the genomes of TR4 strain II5 and VCG 0121, which respectively carry the mating-type loci *MAT*1–1 and *MAT1–2*,share an almost identical chromosomal structure that lacks independent ACs but harbors accessory sequences at the ends of several core chromosomes (Fig. 3, and supplementary Fig. 1). The lack of ACs among these *Foc* genomes was not expected, as ACs were reported in almost all other genomes within FOSC^11,39–41^, including a human pathogenic *F. oxysporum* genome^26^. Classifying VCG 0121 strains as either TR4 or STR4 has been debated. The observation that TR4 strains and VCG 0121 are phylogenetically closely related, causing equally severe disease symptoms in Cavendish bananas, and share this unique chromosome architecture suggests that these strains are potentially offspring of the same mating population, which arose before the recent clonal expansion of TR4 strains, consistent with a recent report^18^.

Lacking distinct ACs, the TR4 genome has its accessory sequences attached to some core chromosomes, as observed among many *F. oxysporum* genomes even with distinct ACs. These sequences share the same properties as distinct ACs, including a higher content of repetitive sequences and lower gene density, and are overrepresented in genes with pathogenesis-related functions. We identified numerous genes encoding *SIX* effectors, the hallmark virulence factor responsible for many Fusarium wilt diseases^10,12,14,38^, and demonstrated for the first time the contribution of *SIX4* to *Foc* virulence using targeted mutagenesis. TR4 accessory genes, especially those expressed during infection of Cavendish bananas, are significantly enriched for genes with known mitochondrial functions. TR4 genes that encode proteins responsible for the mitochondria-localized pathway for fungal NO biosynthesis, as well as fungal NO detoxification functions, are uniquely induced when TR4 enters the banana host, compared to the interactions with R1.

An important signaling molecule, NO homeostasis influences many molecular and physiological processes, including growth development, abiotic and biotic stress response, and signal transduction^42,43^. Fungi NO is a key signaling molecule involved in fungal-plant interactions^34,44–46^ and has been reported in *F. graminearum* to signal host invasion^34^ and in *M. oryzae*^44^ and *Botrytis cinerea*^46,47^ to impose massive nitrosative stress to facilitate disease development. In this study, through a comparative transcriptomics analysis of TR4 (II5) and race 1 (GD02) strains inoculated onto Cavendish bananas, we revealed for the first time that NO is involved in TR4 invasion of banana. Empirically, we confirmed the unique NO induction in TR4 upon JA stimulation. Notably, *SIX4* in Fo5176 induces the Arabidopsis JA biosynthesis pathway^48^ and is highly induced at the early phase of interaction, indicating that this plant hormone is involved in the TR4–banana interaction.

**Figure 6:**
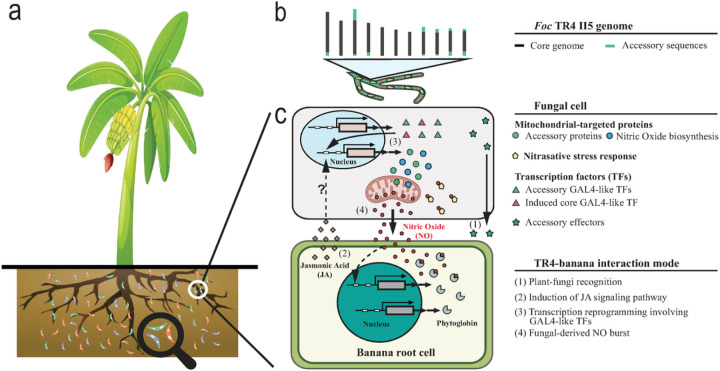
Proposed TR4–Cavendish banana interaction mode. **A.** Spores in the soil represent isolates from three distinct populations, as Pop1 (green), Pop2 (orange) and Pop3 (purple). **B.** The genome of TR4 lacks accessory or pathogenicity chromosomes, reported in all other *F. oxysporum*genomes. Instead, accessory sequences are attached to the ends of core chromosomes. **C.** The proposed TR4-banana interaction model. 1) fungal pathogen-associated molecular patterns and TR4 effectors present at the plant-fungal interface; 2) the recognition of fungal signals activates the plant jasmonic acid (JA) biosynthesis pathway; 3) the increased plant JA hormone stimulates fungal transcription reprograming involving GAL4-like TFs, reoutlining in the induction of the entire mitochondria-localized nitric oxide (NO) biosynthesis pathway and fungal NO detoxification–related genes; 4) the fungal-derived NO burst imposes an enhanced nitrosative and oxidative stress to banana roots.

As TR4 accessory genes are significantly enriched for mitochondrial functions, we hypothesize that the acquisition of these genes allowed TR4 to enhance the nitrosative pressure within the infected banana. We propose the following mode of action behind the TR4–Cavendish banana interaction (Fig. 6). The recognition of fungal signal activates plant defense involving the JA signaling pathway. The JA signal in turn stimulates fungal NO production only on TR4 attributed to the acquisition of TR4-unique accessory genes. With the accompanying up-regulation of NO detoxification genes and increase in fungal detoxification capacity, TR4 creates a NO burst that enhances nitrosative stress within banana roots. The ability to produce a NO burst that both disarms the host defense and protects the fungus from the toxic environment is accomplished through the expansion of TR4-specific accessory genes, including the expansion of transcription factor genes homologous to *FgZC1*, which are known to be involved in fungal NO production and virulence^34^.

Collectively, this study provides insight into the biology of the devastating pathogen TR4 based on a phylogenetic and evolutionary framework.Since first reported in 1989, Fusariumwilt of banana caused by TR4 has continued to spread around the world and now threatens global banana production. Due to its persistence in the soil, one primary strategy is quarantine-based prevention and containment. To avoid future shortages of bananas, scientists around the world must collaborate with farmers and industry partners to establish a sustainable solution. Our initial search for a sustainable solution revealed that the accessory genes carried by the fungal pathogens are not only responsible for producing effector proteins, but also for the elevated NO production within host cells that potentially breaks down host defense. These mechanistic discoveries offer new research directions, such as designing effective NO scavengers for controlling this devastating disease.

## Methods

### Isolate collection, DNA isolation and genome sequencing

Isolates used in this study were provided by Altus Viljoen (Stellenbosch University, South Africa), Randy C. Ploetz (University of Florida, USA) and Chunyu Li (Fruit Tree Research Institute, GDAAS, China). DNA was isolated from freeze-dried mycelium ground in liquid nitrogen as starting material and extracted via three rounds of phenol–chloroform precipitation and treatment with RNase A and proteinase K. Single-molecule real-time (SMRT) sequencing was performed for *Foc* II5, GD02 and CAV045 at the Beijing Genomics Institute (BGI, Shenzhen, China). PacBio libraries were prepared with the SMRTbell template preparation kit (Pacific Biosciences, Menlo Park, CA, U.S.A.) and size-selected at ~20 kb using BluePippin (Sage Science, Beverly, MA, U.S.A.). Sequencing of these libraries generated 5,487 Mb (II5), 7,774 Mb (CAV045) and 11,354 Mb (GD02) filtered data. Additionally, DNA libraries with 500-bp inserts were subsequently constructed for genomic DNA of all 35 isolates. These DNA libraries were 100-bp paired-end sequenced using the Illumina HiSeq 2500 at ~100X coverage, resulting in 5.2–6.7 Gb of sequence data per isolate.

### Genome assembly, gene prediction, annotation and repetitive regions

Genome assembly for Pacbio data was performed using Canu v1.8 ^49^. Data from the Illumina libraries were first trimmed by removing bases with a quality score below 20 at both ends and discarding trimmed reads with lengths less than 70 bp. All sequences in the initial assembly were fed into Quiver along with trimmed Illumina sequences to polish the genome assembly ^50^. *De novo* sequence assembly for isolates with Illumina sequences was conducted using ABySS assembler ^51^. The quality of assembly was evaluated with GRIDSS ^52^ and Sniffles ^53^. All genomic structural variations were checked and corrected manually. Finally, the completion of all assemblies was confirmed using a BUSCO test employing a fungi database (odb9 version) ^54^.

The repetitive sequences in the assembled genomes were identified using RepeatModeler v1.0.7 ^55^ for *de novo* repeats and RepeatMasker v4.0.5 (http://www.repeatmasker.org) for annotations. The output from RepeatModeler was combined with known repeats in *F. oxysporum* 4287 to create the repeat database input for RepeatMasker. Protein-coding genes on the repeat-masked II5 genome assembly were predicted at JGI. Subsequently, the predicted II5 protein-coding genes were used as a training dataset in AUGUSTUS ^56^ to generate the gene predictions for the rest of the *Foc* genomes. Functional annotation (including Pfam and GO terms) for the predicted protein-coding genes was performed by InterProScan, following a standard annotation workflow ^57^. Potential secreted proteins in the II5 genome were predicted using signalP 5.0 with default parameters ^58^. The proteins containing secretory pathway signal peptides were kept. The transmembrane proteins were predicted with TMHMM 2.0 ^59^ and excluded from the secreted proteins. Putative effectors in II5 LS regions were predicted based on the protein size (≤400 amino acid) and the number of cysteine residues (≥4) among the secreted proteins. To find overrepresented GO terms on accessory sequences versus the whole genome, Fisher’s exact test with FDR < 0.05 was performed.

### Read mapping, SNP calling and identification of *MAT1* locus

Trimmed Illumina reads were mapped to the reference genome (*Foc* II5) with Bowtie2 v2.4.2 ^60^. File format conversion was done in the SAMtools v0.1.19 ^61^. Genome Analysis Toolkit (GATK v3.5) was used for SNP calling following the GATK best practice guidelines ^62^. Furthermore, we used vcftools (https://vcftools.github.io) to generate a SNP dataset for phylogenomic analyses. The mating-type locus (*MAT1–1* or *MAT1–2*) was determined for each *Foc* strain by BLASTN (E < 10^-50^) using the *F. graminearum* mating-related genes as queries.

### Phylogenetic analysis and population structure

To determine the phylogenetic framework within the *Foc forma specialis*, nucleotide sequences of 10 conserved single-copy orthologous genes within the *Fusarium* genus were selected and pairwise aligned using ClustalW. After manual removal of regions with poor sequence quality in any isolate, the alignments were concatenated into a single supermatrix. The supermatrix was used as input in MEGA (v10.2.6) to generate a maximum likelihood phylogenetic tree using the general time reversible model with bootstrap test of 500 replicates.

To assess population structure among *Foc* samples, we performed PCA and model-based clustering. PCA was conducted in SNPRelate. The eigenvector and eigenvalues were imported into R for plotting. The model-based clustering program STRUCTURE v2.3.4 was used to analyze population structure of all *Foc* isolates with whole-genome SNPs using the admixture model for 10,000 replications as burn-in ^63^. Structure was run for K values between 1 and 7, and no prior population information was used in the model. The best K value was selected by the Evanno method in STRUCTURE HARVESTER.

### RNA preparation, sequencing and analysis

Tissue cultures derived from Cavendish cv ‘Brazilian’ banana plantlets with four or five leaves (approximately 30 cm in height) were transplanted into Hoagland solution ^64^ and kept in a greenhouse at 25–32°C with a 16-h-light/8-h-dark photoperiod. The wild-type *Foc* TR4 II5 and *Foc* race 1 GD02 were used to inoculate the ‘Brazilian’ bananas at a concentration of 1 × 10^7^ conidia/ml. Three time points (18 HPI for the adsorption stage, 32 HPI for the biotrophic stage and 56 HPI for the necrotrophic stage) were determined by observing the infection process after inoculating banana roots of ‘Brazilian’ with a GFP-tagged *Foc* TR4 II5 transformant, which shares the same growth characteristics and virulence as the wild-type *Foc* TR4 II5. Roots from *Foc* TR4– and race 1–infected plants at 18, 32 and 56 HPI were harvested for total RNA extraction, with three biological replicates per time point. RNA quality and integrity of each library were confirmed with a minimum RNA integrity number (RIN) value of 7. RNA sequencing was done using an Illumina HiSeq 2000 sequencer to generate ~29 million paired-end reads per library with 100-bp read length.

Paired-end RNA-seq reads were assessed for quality by *FastQC* v0.10.1. We used *Trimmomatic* ^65^ to remove poor-quality bases and low-quality reads as well as sequencing adaptors. Leading and trailing read bases with a quality below 20, and a trimmed read length shorter than 70, were removed with parameters LEADING:20, TRAILING:20 and MINLEN:70. The trimmed RNA-seq data were analyzed using STAR ^66^, HTSeq ^67^ and DESeq2 ^68^ pipelines. In short, reads were mapped to reference genomes of *M. acuminata* (DH-Pahang, AA), *Foc* TR4 II5 and *Foc* race 1 GD02 using STAR v2.7.10a. Mapped reads were quantified in HTSeq v0.11.1. Fragments per kilobase of exon per million mapped reads (FPKM) and differential gene expression analysis were conducted using DESeq2 version 1.27.32 with a maximum FDR of 0.05. Genes with per-condition averaged FPKM ≥ 1 were kept in the downstream analysis, and those with an at least 2-fold change in expression were considered differentially expressed between conditions. Corrplot version 0.84 was used to visualize the correlation in gene expression profiles of *Foc* TR4– and race 1–infected plants across the different timepoints at 18, 32 and 56 HPI. The per-condition averaged FPKM values were log-transformed as log_2_(FPKM + 1). Clustering analysis on scaled log-transformed and z-scaled expression values was performed using K-means clustering algorithm "Hartigan-Wong" (R function K-means) and then visualized by ggplot2/3.3.0. This analysis yielded 18 and 24 co-expression gene clusters for fungi and banana, respectively. Gene features with expression correlation with centroids higher than 0.8 were finally accepted by the clusters.

### Gene knockout and complementary mutants

The gene-deletion mutants were generated using the standard one-step gene replacement strategy ^69^. First, the ~1.0 kb of the upstream and downstream sequences flanking the targeted effector genes were PCR amplified, and 3′-terminal (TTGACCTCCACTAGCTCCAGCCAAGCC) and 5′-terminal portion sequences (CAAAGGAATAGAGTAGATGCCGACCG) of the hygromycin resistance cassette were added into the primers of 2R and 3F, respectively, as adapters. Then ∼1.8- and ∼1.9-kb fragments containing the upstream or downstream flanking sequences and the 3′ or 5′ terminus of the hygromycin resistance cassette were amplified by overlap PCR. The above two fragments overlapping by 316 bp were co-transformed into protoplasts of *Foc* TR4 strain II5 (VCG 01213). The complement fragments, which contained the entire effector genes and their native promoter regions, were amplified by PCR and inserted into the modified pFY11 (neomycin resistance) to complement the mutant strains.

### Pathogenicity assays

The pathogenicity assays were performed using a plant inoculation method described by Li et al. (2011). In short, banana plantlets with four or five leaves were individually potted in sterile medium that consisted of three parts vermiculite, one part peat and 0.5 parts coconut coir. Fungal conidia of *Foc* TR4 (VCG 01213), STR4 (VCG 0120), VCG 0120, race 1 (VCG0123) and TR4 II5 mutants were isolated from 3-day-old cultures by filtering through miracloth. Spores were collected, resuspended in sterile water and counted. Twenty plantlets per treatment were inoculated with concentrations of 5,000 conidia/g soil and kept at 25–32°C with a 16-h-light/8-h-dark photoperiod in the greenhouse. The following banana cultivars were used: ‘Zhongjiao No.3’ (Cavendish AAA), ‘Guangfen No.1’ (Pisang Awak ABB) and ‘Fenza’ (Pisang Awak ABB). The disease was scored using a DI from 0–4 (0, no symptoms; 1, some brown spots in the inner rhizome; 2, <25% of the inner rhizome shows browning; 3, up to 75% of the inner rhizome shows browning; and 4, entire inner rhizome and pseudostem are dark brown, dead). Disease index (DI) was calculated using the following equation: DI = [(N_1–5_ × S_1–5_)/(N × S)] × 100%. In this equation, N_1–5_ indicates the number of banana plants with wilt symptoms, S_1–5_ is the value of the score of symptoms, N is the total number of tested banana plants and S is the highest score of symptoms (Cachinero et al., 2002). If the value of DI was 0%, the pathogen lost its virulence completely; if the value was 1–25%, the pathogen had a weak virulence; if the value was 26–50%, the pathogen had moderate virulence; if the value was 51–75%, the pathogen had strong virulence; and if the value was 76–100%, the pathogen was highly virulent.

### Fluorescence assay and confocal microscopy

To assess NO production, *in vivo* fluorescence assays were performed using the NO-sensitive fluorescent dye DAR-4M-AM (cell permeable) as probe. Conidia of each strain were harvested from 3-day cultures, washed by collected as described earlier and resuspended in DI water three times. DAR-4M-AM stock (1 mM) in dimethyl sulfoxide (DMSO) was diluted to 2 µM DAR-4M-AM in 10 mM HEPES (pH 7) on ice. Conidia suspensions were dark-incubated with 2 µM DAR-4M-AM dye for 2 hours at 28°C to allow dye loading, washed twice and resuspended to a spore concentration of 2.5 × 10^5^ spores/ml. The resulting conidia were treated with or without 5 µM MJ for 12 hours at 28°C and visualized in a bright/fluorescence field of view under a confocal microscope at the excitation/emission wavelengths of 544/590 nm. Each experiment contained a minimum of three biological replicates and was repeated independently at least three times. The fluorescence intensity of images was calculated with ImageJ software. Five randomly selected fluorescent areas of each of four images for each treatment were analyzed.

## Supplementary Material

Supplement 1
