# Supplementary material for: Accessory genes in tropical race 4 contributed to the recent resurgence of the devastating disease of Fusarium wilt of banana
Source: Res Sq. 2023 Aug 8:rs.3.rs-3197485. Preprint. [Version 1] doi: 10.21203/rs.3.rs-3197485/v1 (PMC10441461; doi:10.21203/rs.3.rs-3197485/v1)
Supplement: Supplement 1 [file NIHPPRS3197485v1-supplement-1.pdf]

Supplementary Files

This is a list of supplementary files associated with this preprint. Click to download.

- ExtendedDataFig1.pdf
- ExtendedDataFig2.pdf
- ExtendedDataFig3.pdf
- ExtendedDataFig4.pdf
- ExtendedDataFig5.pdf

- ExtendedDataFig6.pdf
- ExtendedDataFig7.pdf
- ExtendedDataFig8.pdf
- ExtendedDataFig9.pdf
- SupplementaryTablesfinalJuly22.xlsx
- Table1final.xlsx
